# CD8^+^ T cell-mediated control of distant tumours following local photodynamic therapy is independent of CD4^+^ T cells and dependent on natural killer cells

**DOI:** 10.1038/sj.bjc.6603792

**Published:** 2007-05-15

**Authors:** E Kabingu, L Vaughan, B Owczarczak, K D Ramsey, S O Gollnick

**Affiliations:** 1 Department of Cell Stress Biology, PDT Center, Roswell Park Cancer Center, Elm and Carlton Sts., Buffalo, NY 14263, USA

**Keywords:** PDT, T cells, tumour immunity, NK cells

## Abstract

Cancer survival rates decrease in the presence of disseminated disease. However, there are few therapies that are effective at eliminating the primary tumour while providing control of distant stage disease. Photodynamic therapy (PDT) is an FDA-approved modality that rapidly eliminates local tumours, resulting in cure of early disease and palliation of advanced disease. Numerous pre-clinical studies have shown that local PDT treatment of tumours enhances anti-tumour immunity. We hypothesised that enhancement of a systemic anti-tumour immune response might control the growth of tumours present outside the treatment field. To test this hypothesis we delivered PDT to subcutaneous (s.c.) tumours of mice bearing both s.c. and lung tumours and monitored the growth of the untreated lung tumours. Our results demonstrate that PDT of murine tumours provided durable inhibition of the growth of untreated lung tumours. The inhibition of the growth of tumours outside the treatment field was tumour-specific and dependent on the presence of CD8^+^ T cells. This inhibition was accompanied by an increase in splenic anti-tumour cytolytic activity and by an increase in CD8^+^ T cell infiltration into untreated tumours. Local PDT treatment led to enhanced anti-tumour immune memory that was evident 40 days after tumour treatment and was independent of CD4^+^ T cells. CD8^+^ T cell control of the growth of lung tumours present outside the treatment field following PDT was dependent upon the presence of natural killer (NK) cells. These results suggest that local PDT treatment of tumours lead to induction of an anti-tumour immune response capable of controlling the growth of tumours outside the treatment field and indicate that this modality has potential in the treatment of distant stage disease.

Cancer survival rates decrease significantly in patients with distant-stage disease; for example, the 5-year relative survival rate is 98% for breast cancer patients with localised disease and only 26% for those with distant-stage disease (Ries *et al*, 2006). There are few effective treatments for distant-stage disease and thus there is an increased interest in therapies that result in both the elimination of primary tumours and the systemic activation of anti-tumour immune responses. Photodynamic therapy (PDT) is an anti-tumour modality that causes tumour destruction through generation of reactive oxygen species (ROS). ROS are produced on illumination of tumours with a specific wavelength of light following administration of a photoreactive drug or photosensitiser. PDT is approved for clinical use in a number of countries, including the United States, for the elimination of early stage malignancies and the palliation of symptoms and the reduction of obstruction in patients with late-stage tumours (Dougherty, 2002; Brown *et al*, 2004).

Photodynamic therapy was initially considered to be primarily a local treatment that caused direct tumour destruction via ROS and indirect tumour damage through vascular damage and induction of inflammation (Oleinick and Evans, 1998; Henderson and Gollnick, 2003). However, several studies have indicated that local PDT treatment of tumours can result in wide-spread effects including systemic neutrophilia (Cecic *et al*, 2001), induction of acute-phase proteins (Cecic *et al*, 2001; Gollnick *et al*, 2003), increased circulating levels of complement proteins (Cecic *et al*, 2006) and systemic release of pro-inflammatory cytokines (Nseyo *et al*, 1990; Ziolkowski *et al*, 1996; de Vree *et al*, 1997; Cecic and Korbelik, 2002; Gollnick *et al*, 2003; Yom *et al*, 2003), all of which indicate the presence of a systemic inflammatory response. Subsequent studies showed that local PDT treatment of murine tumours results in the induction of anti-tumour immunity and resistance to subsequent tumour challenge (reviewed in Canti *et al*, 2002; Castano *et al*, 2006).

The ability of PDT to induce systemic anti-tumour immunity led us to hypothesise that PDT treatment may have an effect on established tumours present outside the local treatment field. To address this hypothesis the effect of PDT treatment of s.c. tumours on the growth of established lung tumours in immuno-competent mice bearing concomitant s.c. and lung tumours was examined. Previous studies that have examined the effect of local PDT on distant tumours have done so in the absence of an intact immune system (Schreiber *et al*, 2002), were unable to detect control of distant disease (van Duijnhoven *et al*, 2003), performed PDT before establishment of distant tumours (Momma *et al*, 1998), or have not discerned a mechanism by which PDT inhibits the growth of tumours outside the treatment field (Gomer *et al*, 1987; Blank *et al*, 2001; Castano *et al*, 2003). In the current study, we demonstrate that control of the growth of tumours present outside the treatment field is dependent upon an intact immune system, is mediated by CD8^+^ T cells and is accompanied by induction of anti-tumour immune memory responses.

It is generally accepted that the generation and maintenance of effective memory CD8^+^ T cells is dependent upon the presence of CD4^+^ T cells (reviewed in Castellino and Germain, 2006), although there are conflicting reports (Marzo *et al*, 2004; Wang *et al*, 2004). We have examined the requirement for CD4^+^ T cells in the generation of anti-tumour immune memory following PDT. In this report, we show that in the absence of CD4^+^ T cells local PDT treatment is able to stimulate the generation of effective and persistent CD8^+^ T cell-mediated immune memory responses and that PDT induction of CD8^+^ T cell-dependent control of distant tumour growth requires natural killer (NK) cells.

These studies demonstrate that PDT induced anti-tumour immunity can be independent of CD4^+^ T cells and suggest that PDT may be beneficial in the control of distant disease.

## MATERIALS AND METHODS

### Animal and tumour models

Eight- to twelve-week-old pathogen-free BALB/cJ, CNCr. 129P2-*Cd40*^*tm1Kik*^*/*J (CD40^−/−^) mice (Jackson Labs, Bar Harbour, ME, USA) or SCID mice (Roswell Park Cancer Institute) were injected s.c. with 3 × 10^5^ EMT6 (murine mammary carcinoma) tumour cells/mouse on the right shoulder (Henderson *et al*, 1985). In experiments examining the effect of PDT on lung tumour growth the mice were injected with 1 × 10^4^ EMT6 tumour cells i.v. to establish tumours in the lungs. The RPCI Institutional Animal Care and Use Committee (IACUC) approved all procedures carried out in this study and the procedures used were in compliance with the UKCCCR guidelines.

EMT6 cells were grown in DMEM, supplemented with 15% FBS and antibiotics (all from GIBCO-BRL, Grand Island, NY, USA); Colon 26 cells were grown in RPMI-1640, supplemented with 10% FBS and antibiotics. All cells were cultured in a humidified atmosphere of 5% CO_2_ in air at 37°C.

### PDT treatment

At day 6 after s.c. tumour inoculation or when the tumours had reached 5 × 5 mm, the mice were injected i.v. with Photofrin® (porfimer sodium; QLT Inc., Vancouver, British Columbia) at a dose of 5 mg kg^−1^. After 18 to 24 h, the primary s.c. tumours were exposed to 630 nm light delivered by an argon laser-pumped dye laser (Spectra Physics, Mountain View, CA, USA) at a total light fluence of 135 J cm^−2^ at a fluence rate of 75 mW cm^−2^. Control groups included mice that were either treated with PDT on a 1 cm^3^ spot of skin opposite the s.c. tumour, had their s.c. tumour surgically removed, were treated with drug or light alone, or received no treatment.

### Assessment of lung tumours

Mice bearing EMT6 lung tumours were euthanised 10–60 days post-PDT and lung tumours were assessed as described earlier (Pulaski *et al*, 1996). Briefly, 1 ml of 15% India ink (diluted in phosphate-buffered saline) was injected via an incision in the trachea. Normal lung tissue absorbs the ink while lung lesions remained white. The lungs were then removed from the rib cage and placed in Fekete's fixative (61% ethanol, 3.2% formaldehyde, 4.1% acetic acid). After a minimum of 2 days in the fixative, lung tumours were counted under a dissecting microscope.

### Cytolytic assay

Spleens were harvested from experimental and control mice 10 days post-treatment, single cell suspensions were generated and depleted of red blood cells. Cells were combined with ^51^Cr-labeled EMT6 or Colon 26 tumour cell and incubated at 37°C in a humidified atmosphere containing 5% CO_2_ for 4 h. Percent specific lysis was calculated as follows: 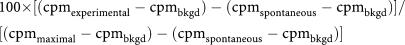


### Flow cytometry

The cell populations present in EMT6 tumours were characterised by fluorescence-activated cell sorting (FACS) analysis, using panels of monoclonal antibodies (MAbs) to detect specific cell surface antigens as described previously (Gollnick *et al*, 1997). Briefly, tumours were disaggregated in 25 ml of HBSS containing 50 mg of collagenase, type II (Worthington Biochemical Corp., Freehold, NJ, USA), and 500 mg of BSA. MAbs conjugated directly with fluorescein or phycoerythrin or biotin was used to quantify cells expressing CD4^+^ and CD8^+^ T-cell antigens (PharMingen, San Diego, CA, USA). Appropriate immunoglobulin isotypes were used as controls. In cases where biotinylated antibodies were used, streptavidin-cychrome (PharMingen) was added as a detection reagent.

For flow cytometric analysis a two-laser FACStar Plus (Becton-Dickinson, San Jose, CA, USA) flow cytometer was used, operating in the ultraviolet (UV) and at 488 nm. Four colours and light scattering properties could be resolved employing 420/20, 530/30, and 575/30 nm band-pass filters and a 640 nm long-pass filter. Data were acquired from 5000 cells, stored in collateral list mode, and analysed using the WinList processing programme (Verity Software House Inc., Topsham, ME, USA). Results are presented as the average percentage of total cells; a total of three animals were analysed for each treatment group.

### Depletion of immune cells

GK1.5 and 53-6.72 rat hybridomas (ATCC, Rockville, MD, USA) were used to generate anti-CD4 and anti-CD8 antibodies, respectively. Briefly, the hybridomas were used to produce ascites in BALB/cJ mice. The ascites were subjected to sodium ammonium sulphate precipitation to obtain CD4 and CD8-specific IgG. Eight- to twelve-week-old BALB/cJ mice were injected with 100 μg of the purified antibodies, a combination of the two antibodies or isotype control (Rat IgG) antibodies (Caltag, San Francisco, CA, USA) for 3 consecutive days, then every other day through the duration of the experiments. After 6 days of depletion, mice were inoculated with s.c. and lung tumours and subjected to PDT as described above. NK cells were depleted by two methods, treatment with anti-asialo-GM1 antibody or treatment with the monoclonal antibody TM-*β*1, which is specific for the IL-2 receptor *β* chain (Tanaka *et al*, 1993). Mice were treated with 50 μl of anti-asialo-GM1 antibody (Wako Chemicals, Richmond, VA, USA) starting on day 1 and continuing every 5 days for the duration of the experiment. TM-*β*1 (100 μg mouse^−1^) was injected into SCID mice 7 days before injection with EMT6 tumour cells. Cell specific depletion was confirmed by flow cytometry at the time of lung harvest.

### Isolation of T-cell subsets

Single-cell suspensions of spleens from naïve mice were prepared and red blood cells were lysed. Monocytes were eliminated by plastic adherence. CD8^+^ and CD4^+^ T cells were isolated by negative selection using MACs beads (Miltenyi Biotech, Auburn, CA, USA) according to the manufacturer's instructions. CD8^+^ T cells were recovered at a >90% purity.

### Reconstitution of SCID mice

Splenocytes harvested at various times following treatment from treated and control mice were depleted of CD8 and/or CD4 expressing T cells (Miltenyi Biotech) and injected i.v. into SCID mice (10^7^ cells mouse^−1^). Two days after reconstitution mice were challenged by injection of 10^4^ EMT6 cells i.v. The presence of lung tumours was assessed 10 days later. For analysis of immune memory responses, purified CD8^+^ T cells (5 × 10^6^ cells mouse^−1^) were injected into SCID mice i.v. Two days after immune cell transfer, the mice were inoculated with EMT6 tumour cells s.c.; 7 days later, tumour bearing recipient mice were treated with PDT and allowed to rest for 40 days. Mice were then challenged with EMT6 tumour cells (10^4^ cells mouse^−1^) i.v. and the presence of lung tumours was assessed 10 days after tumour challenge.

### Statistical analyses

Statistical analysis was performed using a non-paired Student's *t*-test. Significance was defined as *P*<0.05.

## RESULTS

### Local PDT results in systemic control of distant tumours

**Figure 1:**
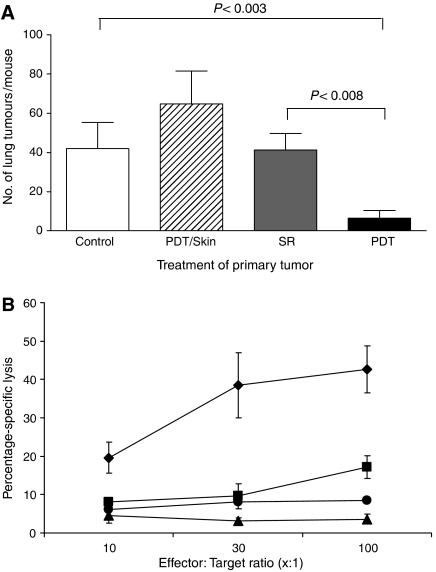
Control of distant disease by PDT is immune cell mediated. (**A**) S.c. tumours present on BALB/cJ mice bearing s.c. and lung tumours were either treated with light only (Control, *n*=5), surgically removed 18–24 h post-Photofrin administration (SR, *n*=16), or received PDT (*n*=16). The PDT/skin column (*n*=6) represents results from mice bearing both s.c. and lung tumours that were treated with Photofrin and had a 1 cm^3^ area of skin on the non-tumour bearing shoulder illuminated with 630 nm light. The average number of lung tumours was determined as described in Materials and Methods; error bars represent s.e.m. Significance was calculated by Student's *t*-test. (**B**) Spleen cells isolated from naïve animals (•) or from mice whose s.c. tumours were surgically removed (▪) were mixed with ^51^Cr-labeled EMT6 target cells at various effector-to-target cell ratios. Spleen cells from mice whose tumours were PDT treated were mixed with either ^51^Cr-labeled EMT6 (□) or Colon 26 (▴) target cells. Effector and target cells were incubated for 4 h and the percent specific lysis was determined as described in Materials and Methods. Spleen cells from a minimum of six mice were used for each point; error bars represent s.e.m.

A number of studies have indicated that PDT enhances anti-tumour immune responses (reviewed in Canti *et al*, 2002; Castano *et al*, 2006) and we hypothesised that the enhanced anti-tumour immunity could lead to control of tumours present outside the PDT treatment site. To determine the existence and extent of such a mechanism, s.c. tumours of mice bearing both s.c. tumours and lung EMT6 tumours were treated with PDT. This led to 90–100% ablation of the s.c. tumour (data not shown) and significantly reduced the number of lung tumours present 10 days post-treatment (PDT, Figure 1A) when compared to the number of lung tumour present in animals whose s.c. tumours were treated with light alone (Control, Figure 1A; *P*<0.003) or whose s.c. tumors were surgically removed (SR; *P*<0.008). In contrast, there was no significant difference between the number of lung tumours present in mice whose s.c. tumours were treated with light alone or surgically removed 18–24 h post-Photofrin administration (*P*>0.56). The inhibition of lung tumour growth observed following PDT was durable; 11 out of 17 mice (62.5%) exhibited no lung tumours 10 days post-PDT, while 12 out of 14 mice (86%) exhibited no lung tumours at 35 days post-PDT and 100% of mice (10 out of 10 mice) were tumour-free at 60 days post-PDT.

We considered that the observed control of lung tumours might have been due to the activation of Photofrin by scattered light from the treatment of the s.c. tumours. To test for this possibility, animals bearing both s.c. and lung tumours were given Photofrin and treated with light 18–24 h later on the shoulder contralateral to the s.c. tumour (Figure 1A; PDT/skin). This treatment did not result in a significant difference in the number of lung tumours when compared to the Control (*P*>0.13). These results support the conclusion that local PDT treatment of s.c. tumours results in a systemic response that leads to control of the growth of tumours outside the treatment field. The data also suggest that treatment of tumour tissue is required for the observed reduction in distant lung tumours.

To demonstrate whether PDT resulted in enhanced cytolytic activity against tumour cells, spleen cells were harvested 10 days post-treatment from animals whose tumours were surgically removed or were treated with PDT and used as effector cells in cytotoxicity assays. As can be seen in Figure 1B, PDT-induced splenic cytolytic activity, which was not observed in naïve animals and was significantly greater than that induced in animals whose tumours were surgically removed (*P*<0.03 at all effector-to-target ratios). Minimal, but significantly greater, cytolytic activity was seen in animals whose tumours were surgically removed as compared to naïve animals at the highest effector-to-target ratio (*P*<0.005). PDT-treated animals did not exhibit significant splenic cytolytic activity against major histocompatibility complex (MHC) matched Colon 26 tumour cells (*P*>0.5 when compared to cytolytic activity present in naïve animals).

To ascertain whether *in vivo* control of tumours present outside the treatment field was also tumour-specific, the s.c. tumours of animals bearing s.c. Colon 26 tumours and EMT6 lung tumours were treated with Photofrin-PDT. PDT of the s.c. Colon 26 tumours had no effect on the growth of EMT6 tumours present in the lung (100.6±34.2 tumours per lung; *P*>0.22 when compared to the number of lung tumours present in animals whose s.c. EMT6 tumours were treated with PDT).

**Figure 2:**
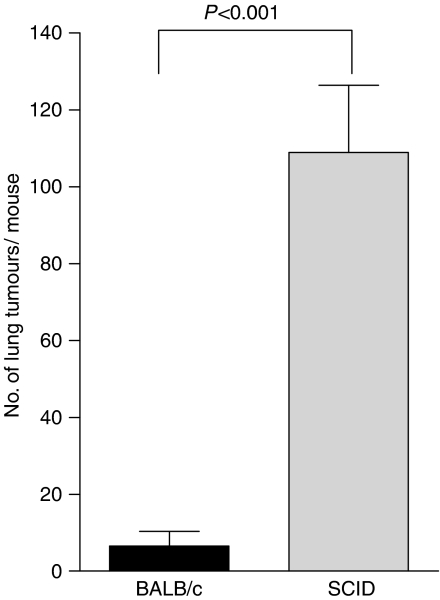
Control of distant tumours by PDT is dependent upon the presence of adaptive immune cells. S.c. tumours present on BALB/cJ or SCID mice bearing s.c. and lung tumours were treated with PDT (BALB/cJ, *n*=16; SCID, *n*=18). The average number of lung tumours was determined as described in Materials and Methods; error bars represent s.e.m. Significance was calculated by Student's *t*-test.

We next established whether the effect of PDT on tumours outside the treatment field was dependent upon adaptive immune cells by examining the ability of PDT to control the growth of tumours present outside the treatment field in immuno-compromised SCID mice, which lack T and B cells. The s.c. tumours of SCID mice bearing s.c. and lung tumours were treated with PDT and the number of EMT6 lung tumours was assessed (Figure 2). The effect of PDT on the inhibition of EMT6 lung tumour growth was significantly reduced in SCID mice (*P*<0.001 when compared to the number of lung tumours present following PDT in BALB/cJ mice).

The combined results of these experiments indicate that PDT is able to control the growth of tumours outside the treatment field in a tumour-specific manner, that PDT enhances tumour-specific cytotoxicity and that control of tumours outside the treatment field by PDT is dependent upon the presence of adaptive immune cells.

### CD8^+^ T cells mediate inhibition of distant tumour growth post-PDT

**Figure 3:**
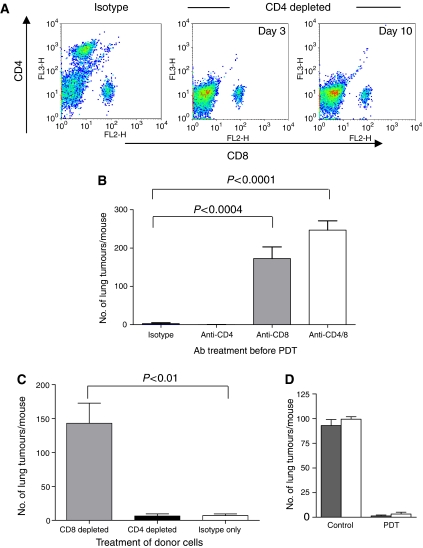
Control of distant disease by PDT is mediated by CD8^+^ T cells. (**A**) BALB/cJ mice were treated with isotype control, anti-CD4 or anti-CD8 monoclonal antibodies. Antibody treated animals were bled and the extent of the depletion was confirmed by flow cytometry. Representative dot plots are shown of samples collected on day 3 and day 10. (**B**) BALB/cJ mice were treated with isotype control (*n*=11), anti-CD4 (*n*=11), anti-CD8 (*n*=10), or a combination of anti-CD4 and anti-CD8 antibodies (*n*=11) before inoculation with EMT6 tumour cells. S.c. EMT6 tumours were treated with Photofrin-PDT (135 J cm^−2^ given at 75 mW cm^−2^) and 10 days post-PDT the average number of lung tumours was determined. (**C**) Splenocytes were harvested from mice depleted of immune cells as in (**B**) 3 days after PDT treatment of s.c. tumours and adoptively transferred into SCID mice (10^7^ cells mouse^−1^). Recipient animals were challenged with 10^4^ EMT6 tumour cells 2 days post-transfer; the presence of lung tumours was assessed 10 days after tumour challenge. (**D**) S.c. EMT6 tumours of BALB/cJ (filled bars) or CD40^-/-^ (open bars) mice bearing s.c. and lung tumours were treated with Photofrin-PDT. Ten days post-PDT the average number of lung tumours was determined as described in Materials and Methods. Each group contains a minimum of five mice. Error bars represent s.e.m. in all figures; significance was calculated by Student's *t*-test.

The results presented in Figure 2 suggest that the ability of local PDT treatment to control the growth of tumours outside the treatment field is mediated by adaptive immune cells as control was lost in immunocompromised mice. To determine which adaptive immune cells were involved in systemic control of tumours following local PDT, BALB/cJ mice were treated with monoclonal antibodies specific for CD4, CD8 or both antibodies together to deplete T-cell subsets before tumour inoculation. The depletion status was maintained for the duration of the experiment as shown in Figure 3A. Depletion of CD4^+^ cells had no effect on the ability of PDT to control tumours present outside the treatment field (Figure 3B; *P*>0.35 when compared to animals treated with isotype control antibodies). In contrast, depletion of CD8^+^ cells or the combination of CD4^+^ and CD8^+^ cells abolished the ability of PDT to control distant lung tumour growth (*P*<0.0004 and *P*<0.0001, respectively, when compared to animals treated with isotype control antibodies). There was no significant difference between the ability of mice depleted of CD8^+^ cells and those depleted of both CD4^+^ and CD8^+^ cells to control distant lung tumour growth. Furthermore, at the time of lung tumour growth assessment all s.c. tumours were regrowing in the PDT-treated mice depleted of CD8^+^ cells and those depleted of both CD8^+^ and CD4^+^ cells; however, the growth of the s.c. tumours was not inhibited in the PDT-treated mice depleted of CD4^+^ cells.

To confirm the importance of CD8^+^ T cells in control of distant disease, SCID mice were reconstituted by adoptive transfer of lymphocytes isolated from EMT6 tumour bearing animals 3 days post-PDT treatment or surgical removal of s.c. tumours. Lymphocytes were treated with either isotype control or depleted of either CD4^+^ or CD8^+^ T cells before transfer into SCID mice. Two days after the adoptive transfer, recipient animals were challenged with 10^4^ EMT6 tumour cells injected i.v. and the presence of lung tumours in the recipient animals was assessed 10 days later. As can be seen in Figure 3C, animals reconstituted with splenocytes depleted of CD8^+^ T cells were unable to transfer tumour growth control to SCID mice; in contrast, splenocytes depleted of CD4^+^ cells were able to provide tumour growth control. In a complementary experiment SCID mice were reconstituted with CD8^+^ T cells isolated from naïve BALB/cJ mice (7 × 10^6^ cells mouse^−1^). Two days after the adoptive transfer of the purified CD8^+^ T cells the recipient mice were inoculated subcutaneously with lung tumours and treated with PDT as above. The presence of lung tumours was assessed 10 days later. SCID mice receiving CD8^+^ T cells had an average of 15.13±11.65 tumours/lung (*n*=9), which was significantly less than the average number of lung tumours present in SCID mice that were not reconstituted (114.6±10.75 tumours/lung, *n*=5; *P*<0.0001). These experiments suggest that control of distant disease following PDT is mediated by CD8^+^ T cells and may be CD4^+^ T cell independent.

To demonstrate further that control of distant disease by PDT was independent of CD4^+^ T cells, we examined the efficacy of PDT in mice lacking CD40. CD4^+^ T cells provide help via secretion of cytokines and CD40/CD40L interactions with dendritic cells (DCs) (Bennett *et al*, 1998; Ridge *et al*, 1998; Schoenberger *et al*, 1998). The control of EMT6 lung tumour growth following PDT treatment of s.c. EMT6 tumours of CD40^-/-^ mice was not significantly different from that observed following treatment of s.c. EMT6 tumours in BALB/cJ mice (Figure 3D). These results support the conclusion that PDT control of tumour growth outside the treatment field is independent of CD4^+^ T cells.

### Local PDT treatment leads to increased infiltration of tumours outside the treatment field by CD8^+^ T cells

**Figure 4:**
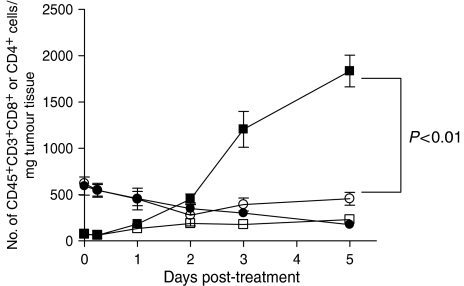
Increased CD8^+^ T-cell infiltrate into distant EMT6 tumours following PDT. The left tumour of mice bearing a tumour on each shoulder was treated with PDT or removed surgically (SR); at various time post-treatment the right untreated tumours were removed and examined by flow cytometry for the presence of CD45^+^, CD3^+^, CD4^+^ cells (circles) or CD45^+^, CD3^+^, CD8^+^ cells (squares) as described previously (Gollnick *et al*, 1997). Results are presented as the number of cells per mg of tumour tissue. Open symbols represent the infiltrate present in untreated tumours when the left tumour was removed surgically; closed symbols represent the infiltrate present in right tumours following treatment of the left tumour with PDT. Tumours from a minimum of three animals were examined from each group. Error bars represent s.e.m.; significance was calculated by Student's *t*-test.

The ability of local PDT treatment to enhance CD8^+^ T-cell control of tumours present outside the treatment field suggests that these cells exhibit increased infiltration into the untreated tumours present outside the treatment field. To test this hypothesis, the degree of T-cell infiltrate into the untreated tumours of mice bearing two s.c. tumours was assessed. The left tumour of mice bearing EMT6 tumours on each shoulder was surgically removed (SR) or treated with PDT (PDT). At various time points following treatment the right tumours were removed and examined by flow cytometry for the presence of infiltrating T cells, which were defined as CD45^+^, CD3^+^, and either CD4^+^ or CD8^+^. The results are shown in Figure 4 and are presented as the total number of CD45^+^, CD3^+^, CD4^+^ or CD8^+^ cells present per gram of tumour tissue. The number of either CD4^+^ or CD8^+^ T cells present in untreated right tumour did not change when the left tumour was surgically removed. There was a significant increase in the number of CD8^+^ T cells present in the untreated right tumour when the left tumour was treated with PDT (*P*<0.01 5 days post-treatment as compared to SR), which was accompanied by a slight decrease in the number of CD4^+^ T cells. Thus local PDT treatment or EMT6 tumours leads to enhanced infiltration of untreated EMT6 tumours by CD8^+^ T cells.

In some mice, right tumour growth was monitored following PDT treatment of the left tumour. PDT treatment of the left tumour led to significant delays in the growth of the right s.c. tumours (data not shown). Castano *et al* (2003) have reported similar findings.

### Local PDT treatment results in the generation of effective memory anti-tumour CD8^+^ T cells in the absence of CD4^+^ T cells

**Figure 5:**
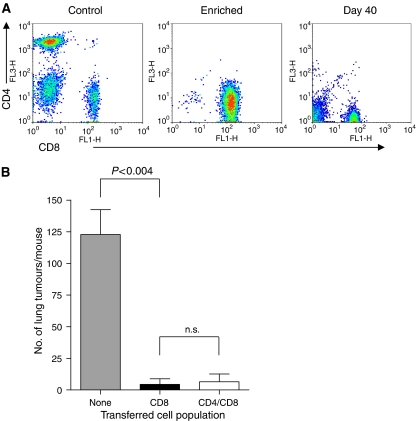
Enhancement of memory CD8^+^ T cells by PDT is independent of CD4^+^ T cells. Spleen cells were isolated from naïve BALB/cJ mice and CD8^+^ or CD 4^+^ T cells were enriched by negative selection as described in Materials and Methods. (**A**) An aliquot of the enriched population was subjected to flow cytometry to assess purity. A representative dot plot from the starting population (Control), the enriched CD8^+^ T-cell population and a recipient mouse after tumour challenge (day 40) is shown. (**B**) The enriched CD8^+^ T cell or combined CD8^+^ and CD4^+^ T cell populations was adoptively transferred into SCID mice (10^7^ mouse^−1^). Recipient mice were inoculated with EMT6 cells and the resulting tumours were treated with Photofrin-PDT. Mice cured of tumours were challenged >40 days post-treatment by i.v. injection of EMT6 cells; 10 days following challenge lungs were examined for the presence of tumours The average number of lung tumours was determined as described in Materials and Methods; error bars represent s.e.m. Significance was calculated by Student's *t*-test.

The results presented in Figure 3 indicate that CD4^+^ T cells were not required for the initial generation of effector CD8^+^ T cells capable of controlling the growth of lung tumours following distant PDT of s.c. tumours. However, CD4^+^ T cells have been implicated in the generation of effective anti-tumour memory CD8^+^ T cells; therefore we tested whether CD4^+^ T cells were critical to the maintenance of the immune response following PDT. SCID mice were reconstituted with purified naive CD8^+^ T cells (Figure 5A). Recipient mice were inoculated with s.c. EMT6 tumour cells 3 days after the adoptive transfer of purified CD8^+^ T cells. The resulting tumours were treated with PDT and the mice were rested for a minimum of 40 days. The rested mice were challenged by intravenous injection with EMT6 tumour cells. Lungs were harvested from the mice 10 days post-challenge and the number of lung tumours present was determined. The transferred CD8^+^ cells were able to prevent lung tumour growth in recipient SCID mice in the absence of CD4^+^ T cells 40 days after PDT (Figure 5B), suggesting that CD4^+^ T cells were not required for the generation of effective memory CD8^+^ T cells.

### NK cells modulate the control of distant tumour growth by local PDT treatment

**Figure 6:**
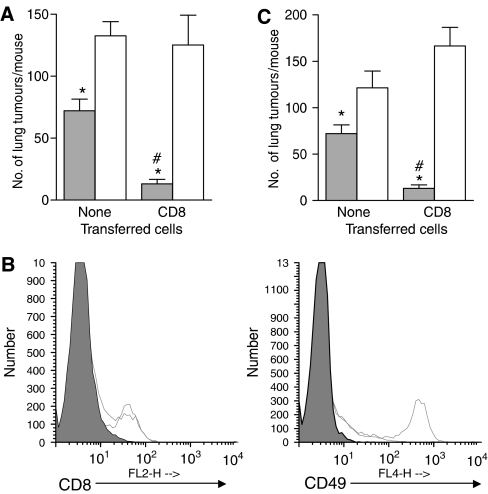
NK cells contribute to control of distant tumours by local PDT. (**A**) SCID mice were treated with TM*β*1 to deplete NK cells as described in Materials and Methods. Five days after TM*β*1 administration, NK-depleted and isotype-treated mice were injected intravenously with PBS or CD8^+^ T cells purified from naïve BALB/cJ mice, followed 2 days later by inoculation of EMT6 tumour cells as described in Materials and Methods. S.c. EMT6 tumours were treated with PDT (135 J cm^−2^ given at 75 mW cm^−2^) and the average number of lung tumours per mouse was determined as described; error bars represent s.e.m. Open bars represent animals treated with TM*β*1; filled bars represent animals treated with isotype control antibodies. Each group contained a minimum of five mice per group. Significance was calculated by Student's *t*-test. ^*^P<0.0007, when the number of lung tumours per mouse treated with TM*β*1 was compared to the number of lung tumours present per mouse treated with isotype control antibody. ^#^*P*<0.0001, when the number of lung tumours per mouse with CD8^+^ T cells and treated with isotype control antibodies was compared to the number of lung tumours per mouse treated with isotype control antibodies. (**B**) Spleen cells were harvested from CD8^+^ recipient mice treated with either TM*β*1 or isotype control antibodies at the time of lung tumour assessment. Cells were stained with antibodies specific for CD3, CD8 and CD49; flow cytometry analysis was performed and cells were gated based on CD3 expression. Representative single colour histograms are shown for cells that were CD3^+^ (left panel) or CD3^-^ (right panel) and expressed either CD8 or CD49 respectively. Filled histograms indicate cells isolated from non-depleted mice stained with isotype control antibodies for anti-CD3 or anti-CD49; open histograms with dashed lines indicate cells isolated from NK depleted mice; open histograms with solid lines indicate cells isolated from non-depleted, isotype treated mice. (**C**) SCID mice were treated with anti-asialo-GM1 to deplete NK cells as described. Depleted and control mice were inoculated with EMT6 tumours as described in Materials and Methods. S.c. EMT6 tumours were treated with PDT (135 J cm^−2^ given at 75 mW cm^−2^) and the average number of lung tumours per mouse was determined as described; error bars represent s.e.m. Open bars represent animals treated with anti-asialo-GM1; filled bars represent animals treated with isotype control antibodies. Each group contained a minimum of five mice per group. Significance was calculated by Student's *t*-test. ^*^P<0.032, when the number of lung tumours per mouse treated with anti-asialo-GM1 was compared to the number of lung tumours present per mouse treated with isotype control antibody. ^#^*P*<0.0001, when the number of lung tumours per mouse with CD8^+^ T cells and treated with isotype control antibodies was compared to the number of lung tumours per mouse treated with isotype control antibodies.

NK cells can mediate CD8^+^ T cell responses in the absence of CD4^+^ T cells (Adam *et al*, 2005; Combe *et al*, 2005). PDT enhances NK activity (Korbelik and Dougherty, 1999) and NK cells have been shown to have an indirect effect on the control of tumour growth by PDT (Hendrzak-Henion *et al*, 1999). To assess whether NK cells contribute to the control of distant tumours by PDT, SCID mice were depleted of NK cells using TM-*β*1, a monoclonal antibody specific for the IL-2 receptor *β* chain (IL-2R*β*), which has been shown to deplete effectively NK cells (Tanaka *et al*, 1993). Mice were injected both subcutaneously and i.v. with EMT6 tumour cells so that s.c. and lung tumours were established. S.c. tumours were treated with PDT in the usual manner. The number of lung tumours per mouse 10 days after PDT of the s.c. tumour of SCID mice bearing s.c. and lung tumours significantly increased in the absence of NK cells (Figure 6A; *P*<0.0007), suggesting that NK cells provide partial control of EMT6 tumours in the absence of T cells. To determine whether NK cells contribute to the CD4^+^ independent ability of CD8^+^ T cells to inhibit the growth of tumours outside the treatment field, 5 days following NK cell depletion, purified naïve CD8^+^ T cells were adoptively transferred into the NK-depleted mice or control mice. In the absence of NK cells, recipient mice treated with PDT were unable to control lung tumour growth even in the presence of CD8^+^ T cells (Figure 6A) and exhibited a significant increase in tumour number (*P*<0.0001). Because activated CD8^+^ T cells express IL-2R*β* it is possible that depletion of NK cells with TM*β*1 results in depletion of the adoptively transferred CD8^+^ T cells. However, as shown in Figure 6B, treatment with TM*β*1 1 week before adoptive transfer of purified CD8^+^ T cells had no effect on CD8^+^ T cell presence. To confirm the results obtained by depletion of NK cells with TM*β*1, NK cell depletion was also carried out by administration of rabbit anti-asialo GM1 (Figure 6C) and similar results were obtained. These studies suggest that NK cells play a role in the control of EMT6 tumours present outside the treatment field and that NK cells are needed for CD8^+^ T-cell control of tumours outside the treatment field.

## DISCUSSION

The results presented here represent the first demonstration of CD8^+^ T cell-dependent control of tumours growing outside the treatment field following PDT; other studies showing inhibition of tumour growth outside the PDT treatment field have not discerned the effector cell type required for control of distant tumours (Gomer *et al*, 1987; Blank *et al*, 2001; Castano *et al*, 2003). We also demonstrate that PDT results in increased CD8^+^ T-cell infiltration into distant untreated tumours and that control of distant tumours is tumour specific. Furthermore, our studies suggest that the control of distant EMT6 tumours is independent of CD4^+^ T cells, but dependent upon NK cells.

Several studies have shown that NK cells can provide help in the establishment of primary and memory CD8^+^ T cell responses. In a study by Adam *et al* (2005) the need for CD4^+^ T helper cells in development of long-term CD8^+^ T-cell memory against A20 lymphoma could be bypassed by NK-DC interaction. Interferon (IFN)-*γ* produced by NK cells was necessary for activation of endogenous DCs and subsequent IL-12 production by the DCs, which led to induction of CTLs. Mocikat *et al* (2003) also demonstrated that NK cells prime DCs to stimulate protective anti-tumour CD8^+^ T cells through secretion of IFN-*γ*. Combe *et al* (2005) showed that NK cells mediate CD8^+^ T-cell immunity against *Toxoplasma gondii* in CD4^+^ T-cell deficient mice in an IL-12-dependent fashion and that depletion of NK cells in mice lacking CD4T cells led to poor CD8^+^ T-cell immunity. The study further showed that NK responses were upregulated in CD4-deficient mice. Hendrzak-Henion *et al* (1999) showed that depletion of NK cells significantly reduced PDT efficacy against EMT6 tumours and suggested that the NK effects were indirect as they were unable to demonstrate direct killing of EMT6 tumour cells by NK cells. These findings, in combination with our present results, suggest that PDT-induced anti-tumour CD8^+^ T-cell immunity may be a result of activation of DCs by NK cells.

Although primary CD8^+^ T-cell responses have been shown to be generated in the absence of CD4^+^ T cells, CD4^+^ T cells appear to be required for the generation of effective anti-tumour memory CD8^+^ T cells in many systems (Belz *et al*, 2002; Janssen *et al*, 2003; Shedlock and Shen, 2003; Sun and Bevan, 2003). The dependence upon CD4^+^ T cells for the establishment of competent memory CD8^+^ T cells varies with the experimental system (Marzo *et al*, 2004; Wang *et al*, 2004; Castellino and Germain, 2006). In our study, effective anti-tumour memory CD8^+^ T-cell responses were observed in mice lacking CD4^+^ cells (Figure 5). These results are supported by a study by Korbelik and Dougherty (1999), in which depletion of CD8^+^ T cells substantially impaired the ability of PDT to suppress EMT6 tumour growth long-term while depletion of CD4^+^ T cells had a minimal effect. However, it is possible that the transferred naïve CD8^+^ T-cell population used to study the memory response (Figure 5), which was >95% pure, contained sufficient CD4^+^ cells to provide help for the maintenance of the memory CD8^+^ T-cell population.

The ability of local PDT treatment of tumours to enhance the formation of memory CD8^+^ T cells in the absence of CD4^+^ T cells is in agreement with studies showing that DC stimulated *in vitro* with CD40 cross-linking antibodies (Ridge *et al*, 1998; Ribas *et al*, 2001) or toll-like receptor (TLR) ligands (Bullock and Yagita, 2005; Shi and Ziang, 2006) and injected into mice could lead to the formation of memory CD8^+^ T cells. However, our findings contrast with recent findings in viral (Belz *et al*, 2002; Janssen *et al*, 2003; Shedlock and Shen, 2003) and bacterial (Sun and Bevan, 2003) systems in which generation of memory CD8^+^ T cells was found to be dependent upon the presence of CD4^+^ T cells. One potential explanation for the discrepancy lies in the maintenance of the memory CD8^+^ T cells. Bevan and colleagues have shown that although memory CD8^+^ T cells could be generated in the absence of CD4^+^ T cells, their numbers and the functionality of the memory cells diminished over time (Sun and Bevan, 2003; Sun *et al*, 2004) and that diminished function was apparent 30 days post-activation (Sun *et al*, 2004). Our studies examining CD8^+^ T cell function 40 days post-activation did not reveal a deficiency, however; it is possible that CD8^+^ T-cell function could be diminished at later time points.

Depletion of CD4^+^ cells by treatment with anti-CD4 antibodies will also result in depletion of T helper cells and regulatory T cells, which are CD4^+^ and have been shown to suppress anti-tumour immunity (reviewed in Zou, 2006). Thus it is possible that our results showing control of distant disease following depletion of CD4^+^ cells or transfer of purified CD8^+^ T cells is a result of enhanced CD8^+^ T-cell activity in the absence of suppression. However, depletion of CD4^+^ cells in immuno-competent mice does not significantly enhance the ability of CD8^+^ cells to control distant disease (Figure 1B) and the ability of CD8^+^ cells to control distant tumour growth is not significantly affected by the presence of CD4^+^ T cells when purified cell populations are transferred to immunocompromised mice (Figure 5B).

Establishment of CD8^+^ T-cell memory responses in the absence of CD4^+^ T cells also appears to be related to the extent and nature of the inflammatory response generated at the time of antigen exposure, which can affect the activation status of antigen-presenting cells (Matzinger, 1994; Kaisho and Akira, 2003; Bullock and Yagita, 2005). The activation status of antigen-presenting cells, particularly that of DCs is critical to their ability to stimulate fully the generation of CD8 memory T cells (Ridge *et al*, 1998; van Mierlo *et al*, 2004). DC activation or licensing by CD4^+^ T cells can be replaced by DC recognition of exogenous (Janeway and Medzhitov, 2002) or endogenous (Matzinger, 2002) TLR ligands or danger signals. Endogenous danger signals include release of stress proteins and products of dead cells including heat shock proteins (HSP) and uric acid (Moseley, 2000; Gallucci and Matzinger, 2001; Shi *et al*, 2003). PDT causes systemic inflammation and direct tumour cell death (Dougherty *et al*, 1998; Castano *et al*, 2006) that is accompanied by cell surface expression of HSP70 (Korbelik *et al*, 2005). Thus it is possible that local PDT treatment of tumours leads to release of factors capable of bypassing the need for CD4^+^ T cells in the activation of DCs. In support of this hypothesis, we have recently shown that PDT treatment of EMT6 and Colon26 tumours enhances maturation and activation of DCs and that DCs isolated from PDT treated mice are able to stimulate T-cell effector functions (Gollnick *et al*, 2006).

Diverse PDT regimens and photosensitisers can lead to differing levels of inflammation (Henderson *et al*, 2004) and it has been suggested that the development of an adaptive immune response is dependent upon the degree of the inflammatory response (Akira *et al*, 2001; Lanzavecchia and Sallusto, 2001; Nestle *et al*, 2001; Schnare *et al*, 2001; Matzinger, 2002). The regimen-dependent induction of inflammatory responses following PDT could also account for the failure of other groups to detect an effect of Foscan (*meta*-tetrahydroxyphenylchlorin)-PDT of orthotopic rat liver tumours on liver tumours present outside the treatment field (van Duijnhoven *et al*, 2003). In addition, NK cell activation of DCs in anti-tumour immunity appears to depend upon tumour expression of NKG2D ligands (Adam *et al*, 2005), or expression of costimulatory molecules such as CD70, CD80 or CD86 (Kelly *et al*, 2002); EMT6 tumours are immunogenic (Siemann *et al*, 1981) respond well to PDT (Korbelik and Dougherty, 1999) and may be more susceptible to immune-mediated control. PDT is likely to be less effective at stimulating systemic anti-tumour immune responses against less immunogenic tumours such as those used in other PDT studies of systemic immunity (van Duijnhoven *et al*, 2003). Thus PDT control of distant disease is likely to be regimen and tumour-dependent.

The perceived lack of systemic effectiveness of PDT has limited its usage for the treatment of advanced disseminated disease. The findings presented in this study suggest that it may be possible to devise clinical PDT treatment regimens capable of enhancing systemic anti-tumour immune responses. Most of the therapies currently used to treat disseminated disease, including chemotherapy and radiation, have negative effects on the host immune response. The availability of a tumour treatment capable of eliminating the primary tumour while simultaneously stimulating anti-tumour immunity would be significant.
